# Immuno-MALDI-MS in Human Plasma and On-Chip Biomarker Characterizations at the Femtomole Level

**DOI:** 10.3390/s121115119

**Published:** 2012-11-06

**Authors:** Alain Rouleau, Marven El Osta, Géraldine Lucchi, Patrick Ducoroy, Wilfrid Boireau

**Affiliations:** 1 Institut FEMTO-ST, Université de Franche Comté, CLIPP (Clinical-Innovation Proteomic Platform), 25044 Besançon, France; E-Mail: alain.rouleau@femto-st.fr; 2 SFR Santé STIC, CLIPP (Clinical-Innovation Proteomic Platform), Université de Bourgogne, Centre Hospitalier Universitaire de Dijon, 21000 Dijon, France; E-Mails: marven.elosta@clipproteomic.fr (M.E.O.); geraldine.lucchi@clipproteomic.fr (G.L.); patrick.ducoroy@clipproteomic.fr (P.D.)

**Keywords:** Surface Plasmon Resonance, mass spectrometry, immuno MALDI-MS, biomarker, proteomics

## Abstract

Immuno-SPR-MS is the combination of immuno-sensors in biochip format with mass spectrometry. This association of instrumentation allows the detection and the quantification of proteins of interest by SPR and their molecular characterization by additional MS analysis. However, two major bottlenecks must be overcome for a wide diffusion of the SPR-MS analytical platform: (i) To warrant all the potentialities of MS, an enzymatic digestion step must be developed taking into account the spot formats on the biochip and (ii) the biological relevancy of such an analytical solution requires that biosensing must be performed in complex media. In this study, we developed a procedure for the detection and the characterization at ∼1 μg/mL of the LAG3 protein spiked in human plasma. The analytical performances of this new method was established, particularly its specificity (S/N > 9) and sensitivity (100% of LAG3 identification with high significant mascot score >68 at the femtomole level). The collective and automated on-chip MALDI-MS imaging and analysis based on peptidic fragments opens numerous applications in the fields of proteomics and diagnosis.

## 1. Introduction

Immuno-Mass Spectrometry is an emerging technique which is based on affinity enrichment on functionalized solid phases coupled with matrix-assisted laser desorption ionization time of flight mass spectrometry (MALDI-MS). This approach has a great potential for the elucidation of protein complexes, the identification of unknown partners and the characterization of biomarkers [1]. Indeed the hyphenation of immunoassays with mass spectrometry appears to be very promising in allowing qualitative or quantitative measurements with the possibility to analyze part of the sequence of captured proteins [2,3].

Among the different strategies currently being developed, the combination of MALDI-MS with surface plasmon resonance (SPR) wherein SPR is employed to monitor Ab-Ag interaction and protein tracking in real time is of major interest [4–8]. Since 2000, SPR-MS has been performed following two analytical pathways: the off-chip approach, which is based on the elution of bound analytes prior to mass spectrometry analysis and the on-chip approach, which benefits from *in-situ* arrays analysis provided by MALDI-TOF-MS spectrometers. This latest technology has been successfully validated for the sensitive detection of various analytes in ideal solutions and their identification by MS and MS/MS [9–11]. However results presented in these previous SPR-MS studies were obtained after manual (bio)chemical treatments prior to MS analysis which is incompatible with clinical studies (which required very good reproducibility and a very low technical variation only provided by validated automation procedures). Until now, a major breakthrough still remaining is that of achieving “on-chip” detection and characterization of protein biomarkers present in biological samples such as human plasma. Such an issue implies that non-specific adsorption must be controlled in order to obtain significant SPR measurements after analyte fishing, but also to avoid non-specific peptidic signals in the mass spectra which could bias the characterization and the identification of biomarkers. To conclude, it is crucial that this instrumental combination through immunochips does not alter the intrinsic performance of each instrument used separately. We have recently established an analytical platform called SUPRA-MS which achieves these goals in a macroarray format [12].

In this paper, the presentation of the chip-MS coupling technique to the broader SPR scientific community by using Biacore SPR instrumentation based on immunochips and a lateral flow device in four distinct channels is proposed. Validation of the procedure is obtained in using the LAG3 protein, a potential marker of human breast cancer and tuberculosis, [13], which was spiked in known amounts in human plasma. Real-time detection of LAG3 in plasma was monitored and followed by an automated protocol of collective chemical and biochemical treatments prior to mass spectrometry analysis. Thus we obtained significant identifications of LAG3 by peptide mass fingerprints (PMFs) and MS/MS analysis at the femtomole level that open the way to the more qualitative characterization of targeted proteins after their detection on Biacore biochips.

## 2. Experimental Section

### 2.1. Materials

Mercapto-1-undecanol (MUOH), mercapto-1-hexadecanoic acid (MHA), N-hydroxysuccinimide (NHS), tris(2-carboxyethyl)phosphine (TCEP), acetonitrile, NH_4_HCO_3_, and rat serum albumin (RSA) were purchased from Sigma–Aldrich (Saint-Quentin Fallavier, France). α-RSA antibody (Sheep IgG, ref:0220-2424) was purchased from AbD Serotec (division of MorphoSys, Oxford, UK). Digest from bovine serum albumin (BSA) and α-cyano-4-hydroxycinnamic acid (HCCA) were purchased from Bruker Daltonics (Bremen, Germany). N-(3-Dimethylaminopropyl)-N-ethyl-carbodiimide (EDC), N-hydroxysuccinimide (NHS) and ethanolamine (HCl, pH 8.5, 1 M) were purchased from Biacore (GE Healthcare, Uppsala, Sweden). Protein Lymphocyte Activation Gene 3 (LAG3) and α-LAG3 (Mouse IgG2A) were provided by Immutep SA (Châtenay Malabry, France). The running buffer was phosphate buffered saline (PBS), 10 mM at pH 7.4 with NaCl (138 mM), KCl (2.7 mM) and Tween 20 (0.05%).

For protein digestion, we used trypsin enzyme (Gold Mass Spectrometry Grade from Promega) in sodium acetate buffer at 10 mM. All of the buffers were prepared using ultrapure water (18 MΩ/cm resistance, Purelab prima from Elga LabWater, Antony, France). Human apheresis plasma treated with Methylene Blue was kindly provided by the National Blood Transfusion Center (Bourgogne Franche-Comté, France).

### 2.2. Chemical Functionalization and Realisation of Immuno-Chip

Our homemade chips were chemically functionalized with a self-assembled monolayer composed of a mixture of MUOH and MHA at 1 mM (97/3 by mole). The sensor chips were cleaned with absolute ethanol (Carlo Erba: Val de Reuil, France) then treated overnight and rinsed with ultra pure ethanol and water (Elga LabWater).

Afterwards, in the Biacore 2000 (Biacore, GE Healthcare), carboxyl groups of MHA are activated for 7 minutes at 10 μL/min by two injections of a solution of 100 mM NHS and of 400 mM EDC. The immunochips were realised by the immobilization of the monoclonal antibody A9H12 (Immutep SA) that specifically recognizes LAG3 recombinant protein used for this study and the control antibody is an α-RSA polyclonal antibody. The antibodies are diluted to 40 μg/mL in 10mM sodium acetate buffer (pH 5.2) and injected at 2 μL/min. At last, the free NHS sites were deactivated by one injection for 14 minutes at 10 μL/min with a solution of 1 M ethanolamine HCl. The running buffer used for the immobilization was PBS (10 mM at pH 7.4 with NaCl 138 mM, KCl 2.7 mM)

### 2.3. SPR Analysis

Capture of LAG3 added in total human plasma was performed at 25 °C at a flow rate of 20 μL/min for 15 minutes. The running buffer used was PBS, 10 mM with Tween 20 (0.05%). At the end of the interaction, the chip was removed from the apparatus following the “undock procedure” of the Biacore2000 apparatus and rinsed with ultra pure water to be analyzed by mass spectrometry.

### 2.4. On-Chip Reduction and Tryptic Digestion

The treatments of the captured proteins were performed directly on chip using ImagePrep Station (Bruker Daltonics, Bremen, Germany). Using a pinhole sheet that vibrates at supersonic speed triggered by a piezoelectric actuator, it is possible to generate a fine spray of solutions onto a MALDI target surface in a nitrogen atmosphere. The size of the droplets is controlled by the power of the spray. To avoid spot-to-spot cross contamination, the duration and the frequency of the spray were selected to maintain deposition of small droplets in short spray cycles thus preventing droplet confluence and analyte delocalization.

The treatment through the ImagePrep of the captured proteins prior MALDI-MS analysis includes the following steps: reduction step (TCEP 10 mM in 0.1 M NH_4_HCO_3_, Sigma Aldrich) followed by a tryptic digestion step (10–40 ng/μL) both at 37 °C and the matrix deposition step (HCCA (1 mg/mL) in 50/50 v/v water/acetonitrile with 0.25%TFA) at room temperature. The alkylation step isn't necessary because of the short digestion time which reduces the risk of disulfide bonds formation from free sulfhydryl groups [14]. Each step was optimized to obtain adequate layers to ensure homogeneous coverage and optimal efficiency: the different steps were validated on small amounts (in accordance with our previous paper, *i.e.*, in the range 5–15 femtomoles/mm^2^ [10]) of known proteins deposited on the biochips. Selections of the best conditions and establishment of the global procedure are based on the number of detected peptides, the number of matched peptides and the intensity of peptides used to identify the model proteins.

### 2.5. On-Chip MS Analysis

The biochip was placed in the UltrafleXtreme MALDI-TOF/TOF (Bruker Daltonics, Bremen, Germany) using a prototype adapter target designed for biochips. The UltrafleXtreme MALDI was equipped with a smartbeam-II laser (1,000 Hz repetition rate), which enables ultra-high data acquisition speed in both MS and MS/MS. The UltrafleXtreme MALDI-TOF MS was operated in reflector positive ion mode for the mass measurements of peptides resulting from the on-chip digestion. MS spectra were automatically acquired using an AutoXecute method combined with Fleximaging 2.1 software (Bruker Daltonics) that generates images of peptide distribution across the biochip's surface with a range of ±0.5 Da as defined in the mass filtering parameters in the software. The biochip's surface was analyzed with a raster of 120 μm. Each mass spectrum resulted from an accumulation of 2000 laser shots in a random mode across each position, with an acceleration voltage of 25 kV and a pulsed ion extraction of 80 ns. MS/MS spectra were acquired manually, with the source acceleration voltage set at 7.5 kV and a pulsed ion extraction of 60 ns. Each MS/MS spectrum obtained, resulted from an average of 1000 laser shots in the parent mode and 2000 laser shots in the fragment mode.

A local Mascot server (Mascot version 2.2.01; Matrix Science, Boston, MA, USA) and the Swiss-Prot TrEMBL database was used for protein identification based on MS or MS/MS spectra with the following parameters: Mammals, trypsin digestion and one missed cleavage site. The mass tolerance in the MS mode was set at 50 ppm. Mass tolerances of fragments in the MS/MS mode were set at 0.6 Da.

## 3. Results and Discussion

### 3.1. SPR Analysis in Human Plasma

The IgG grafting procedure has been previously published [10]. Briefly, we functionalized a homemade gold chip with a mixture of MUOH and MHA at 97/3 by molar ratio, respectively. Activation of the carboxylic moiety was performed with NHS and EDC. Immobilization of IgGs (here α-LAG3 and α-RSA) was achieved through primary amine group of lysine and was monitored in real time with SPR (Biacore) measurements. Finally, inactivation of the chip was obtained with a pulse of ethanolamine. High control on the surface coverage was performed according to the time of injection and the concentration of various IgGs and we obtained routinely immobilization levels comprised between 5 and 15 fmole/mm^2^. This procedure does not yield a preferential orientation of IgGs at the surface of the chip in comparison with proteins A or G strategies, nevertheless as previously demonstrated, we obtained equilibrium states of biomolecular recognitions between LAG3 and α-LAG3 for a molecular ratio of 1/1 [15].

**Figure 1.**
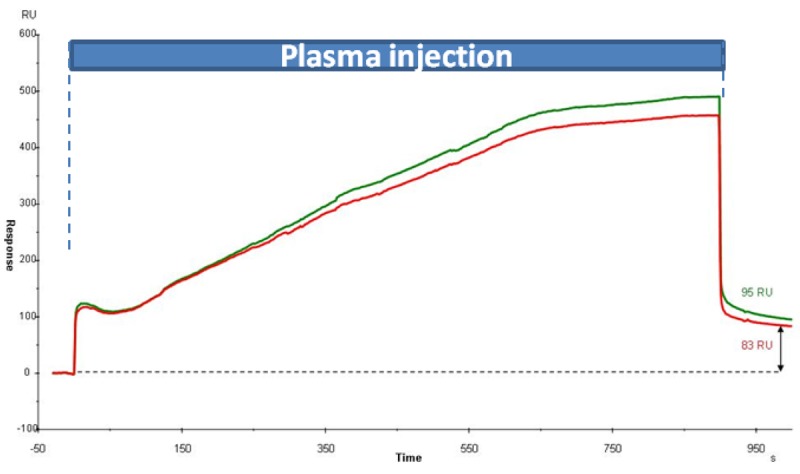
Sensorgram of the response of diluted human plasma (2.5%) onto immunochips, especially α-LAG3 (green curve) and α-RSA (red curve) at 25 °C, during 15 min at a flow rate of 20 μL/min.

These immunochips were exposed to human plasma. Even without treatment of the biochip surface with blocking agents (such as mammalian albumin), the observation of specific antigen-mAb binding was achieved from total human plasma diluted by 40 in PBS-Tween. Results of non specific adsorption to IgGs̵ monolayers are illustrated in Figure 1. They show that unspecific binding of molecules in plasma to the mAbs has slightly increased and was saturated to 800 s during plasma injection. However, unspecific binders were removed quickly by the running buffer (900 s) indicating only weak K_D_s. For each plasma injection we observed that the level of non-specific adsorption is maintained below 150 RU on all our experiments, *i.e.*, below 150 μg/mm^2^, without cleaning procedure nor the use of blocking agents.

**Figure 2.**
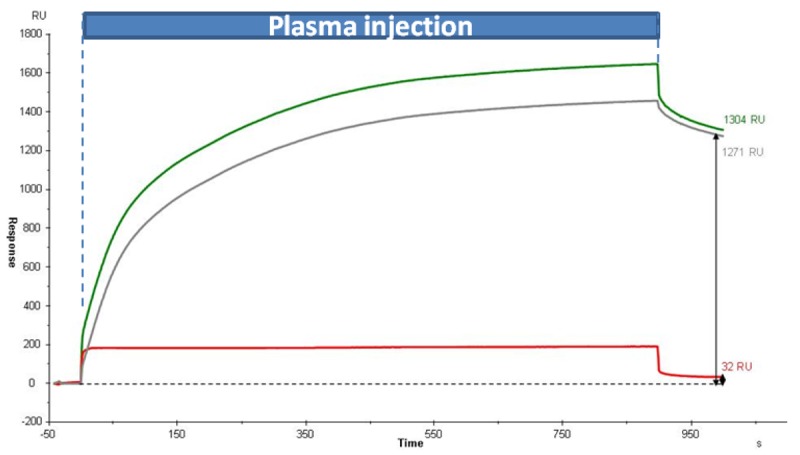
Sensorgram of interaction between LAG3 capture in diluted human plasma (2.5%) spiked with LAG3 (12.5 nM) with α-LAG3 (green curve) and α-RSA (in red) biochip surface. Differential of the response was indicated by the grey curve.

The protein LAG3 was spiked in human plasma at a low concentration, ∼12.5 nM. At this concentration, LAG3 species represent less than 0.1% of total protein of the biological samples, *i.e.*, 2 μg/mL in diluted human plasma (2.5%). Sensorgrams of the interactions in Figure 2 showed the specific capture of LAG3 (green curve) and that LAG3 binding was saturated after few minutes with an equimolecular Ab/Ag ratio (8.8 fmoles LAG3 captured by 9.6 fmoles immobilized α-LAG3) demonstrating the good efficiency of the immunochip in complex media. Under these conditions, we observed a significant S/N ratio (≥40); the differential response was represented in grey.

### 3.2. Automation of the Tryptic On-Chip Digestion and Matrix Deposition Prior to MS

#### 3.2.1. Sensitivity—Automation of Chemical Matrix Deposition

Manual dispensing of (bio)-chemical solutions is incompatible with the geometry of the four arrays of a Biacore chip which are too close together to allow individual treatments without avoiding cross contamination. It was therefore decided to use a spray-type approach which has been developed to treat biological tissue sections prior to mass spectrometry analyses. The benefits of robotic sample processing are the reproducibility, traceability and the ability to use a confined space where the parameters that change the quality of crystallization are controlled. To validate the collective processing of immunochip using a spray, several criteria were defined including: sensitivity, reproducibility, uniformity and resolution. The protocols had to be optimized to analyze the biochip on which a protein monolayer was reconstituted. An initial study has shown the potential of hyphenated SPR-MS to identify and characterize LAG3 protein captured from a simple buffered solution after a manual procedure at the 5 to 10 fmole/mm^2^ level [10]. In spreading in droplets 5 to 10 fmoles of BSA digest in an area equivalent to 1 mm^2^, the surface coverage obtained routinely during protein immobilization and/or interaction in the Biacore instrumentation is mimicked.

**Figure 3.**
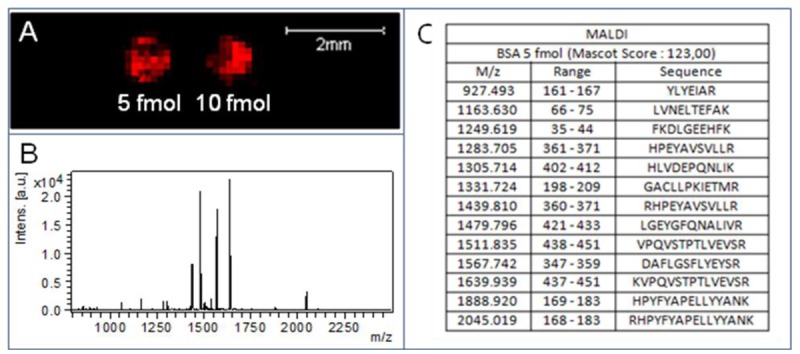
on chip MALDI-MS imaging and analysis of a BSA digest after matrix deposition by Imageprep. (**A**) The distribution of a specific tryptic BSA peptide (*m/z* 1487.79) is shown in red. (**B**) MS spectrum obtained on one laser position in the 5 fmol BSA digest spot identifying Bovin Serum Albumin with a Mascot Score of 123.00 and 13 specific peptides (**C**).

Figure 3 shows that the optimized HCCA matrix deposition procedure described in the Experimental allows one to obtain results in phase with the constraints related to the combined SPR-MS approach: good on-chip sensitivity which allow significant BSA identification at the femtomole level and no peptide delocalization after spot treatments which give confidence to array analysis.

#### 3.2.2. Automation of Bio-Chemical Treatments Prior MS

The validation of the automation step of chemical matrix deposition obtained in Section 3.2.1 is a necessary but not sufficient procedure for exploiting all the potentialities of MALDI MS analysis. If the use of spray has been previously tested for matrix deposition on spotted chip and has lead to mass spectra of entire proteins as transferrin and b-2-microglobulin [16] until now its spreading remains limited for deep protein characterizations. A major drawback is that to perform fine characterization of proteins by MS, it is necessary to analyze their fragments (resulting from enzymatic digestion) instead of working their whole structure. The establishment of an automated protocol of on-chip digestion which preserved the pattern of spots is an important breakthrough. To achieve this, the enzyme was sprayed on the surface of the biochip using the Imageprep to perform an *in situ* digestion. To optimize the efficiency of the enzymatic digestion, two parameters were modulated: the concentration of trypsin and the temperature. The Imageprep work usually at room temperature but we experiment spray cycles at 37 °C. The criteria used to optimize the digestion process are: the intensity of the peaks, the number of specific peptides of the targeted protein and the total number of peptides.

**Figure 4.**
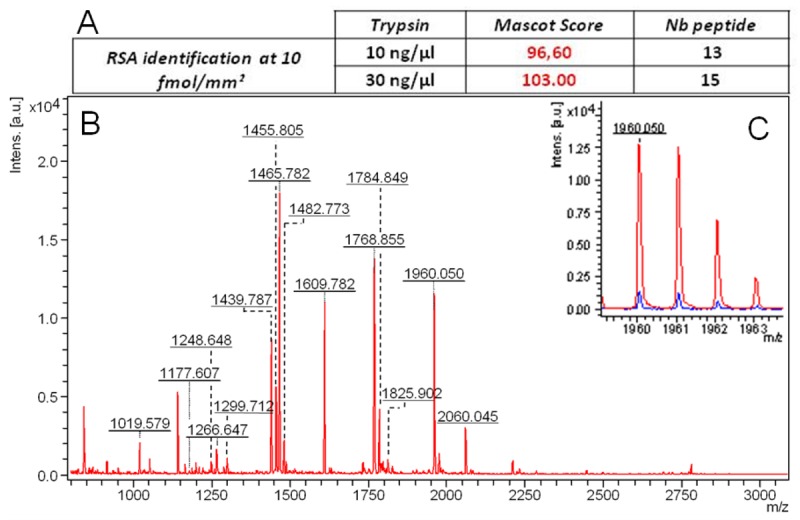
Mascot Identification score obtained and the number of peptides detected after reduction and digestion of the deposited RSA at 10 fmol/mm^2^ using 10 or 30 ng/μL of trypsin (**A**). MS spectrum of RSA after digestion by the solution containing 30 ng/μL of trypsin (**B**) Difference in intensity of a specific RSA peak (1,960.05 *m/z*) between 10 ng/μL (blue spectrum) and 30 ng/μL (red spectrum) conditions (**C**).

Figure 4 shows that the concentration of 30 ng/μL of trypsin sprayed is more efficient and gives an identification score of 103, with the highest [15] number of the specific RSA peptides detected. Figure 4(C) shows the intensity difference obtained between both 10 ng/μL and 30 ng/μL conditions for peptide 1960050. The sequence coverage of the matching peptides detected after reduction and digestion of the deposited RSA at 10 fmol/mm^2^ using 30 ng/μL of trypsin reaches 23% (see Appendix 1). The tests performed with higher concentration of trypsin (>40 ng/mL) are not conclusive because autolysis peptides of the enzyme become abundant and decrease the specific signal of the analyte (data not shown).

#### 3.2.3. Reproducibility—Automation of Chemical Matrix Deposition and On-Chip Digestion

**Figure 5.**
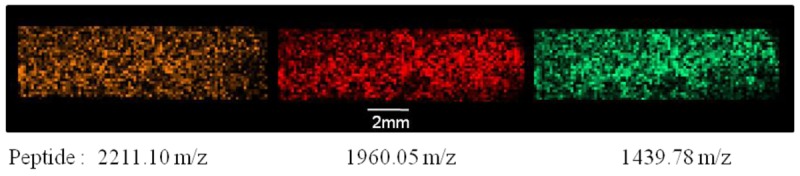
Peptide distribution 2211.10 (specific to trypsin), 1960.05 and 1439.78 (specific to RSA), after analysis of a 10 fmol/mm^2^ RSA layer digested by trypsin at 30ng/μl with the deposition of HCCA matrix at 1 mg/mL.

The automation of procedures through the ImagePrep will also help reducing technical variability and allow us to obtain more robust results. The procedure used to treat the sample is the same as the manual one which contains a step of reduction and digestion. Figure 5 shows the homogeneous deposit of trypsin in imaging the location of a specific peptide from trypsin (2,211.10 *m/z*) and two specific peptides from RSA (1,960.05 and 1,439.78 *m/z*). The homogeneous deposition of the enzyme provides good intrabiochip reproducibility, but also allows treating up to four biochips simultaneously, thus ensuring a comparable analysis between biochips which have been exposed to different types of biological samples. The good reproducibility is also provided by the controlled atmosphere of the ImagePrep which prevents the influence of outside temperature and the hydrometric degree onto the enzymatic activity and the speed of matrix crystallization.

### 3.3. Experiments of SPR-MS in Human Plasma

**Figure 6.**
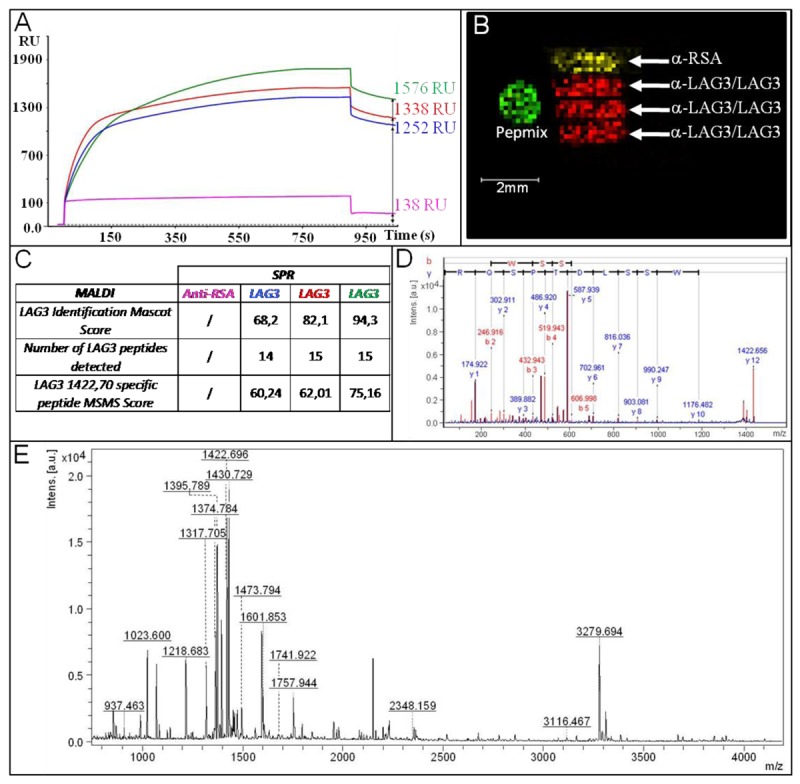
(**A**) Sensorgram of LAG3 capture in diluted total human plasma (2.5%) on α-LAG3 (in green, red and blue) and α-RSA (in pink). (**B**) On-chip MALDI-MS image showing the distribution of the specific peptides from LAG3 (*m/z* 1,422.69) in red, α-RSA (*m/z* 1,697.20) in yellow and calibration sample (Pepmix, *m/z* 1,296.68) in green. (**C**) Results of on-chip MALDI-MS and MS/MS analysis. (**D**) MS/MS spectrum obtained for the peak *m/z* 1,422.69. (**E**) On-chip MALDI-MS spectrum of LAG3 after *in situ* tryptic digestion.

As illustrated in the SPR part, we performed LAG-3 tracking in human plasma with the help of Biacore instrumentation. In this SPR-MS experiment we decided to explore the entire spot configuration provided by the fluidic cartridge of Biacore's instrumentation, *i.e.*, four parallel arrays of 1.2 mm^2^ spaced of 200 μm each. The first three spots were bio-functionalized with α-LAG in a coverage window comprise between 7.7 and 9.2 fmoles/mm^2^ and the fourth with the same amount of α-RSA (7.9 fmoles/mm^2^). After deactivating the surface with ethanolamine, the biochip has been exposed to human plasma (2.5%) spiked with LAG3 (12.5 nM). As already showed in Figure 2, the capture of LAG3 in a reduced time of 15 min was reproducible, specific and efficient with a S/N > 9 (Figure 6(A)). At this level of capture (steady state of biomolecular interaction analysis), we stopped the SPR experiments and removed the chip from Biacore 2000 apparatus through the “undock procedure”.

The condition of reduction and digestion were based on the result from optimization experiments (Section 3.2). Briefly, the optimized conditions are a reduction step (TCEP 10 mM in 0.1 M NH_4_HCO_3_,) followed by a tryptic digestion step (30 ng/μL) and the matrix deposition step (HCCA (1 mg/mL) at 23 °C. The efficiency of tryptic digestion on the surface and the homogeneity of the chemical matrix used for MALDI-MS experiments, allowed the detection of at least 14 specific peptides of the LAG3 protein in the 900 to 4,000 *m/z* range (Figure 6(C,E)). Identification of LAG3 (LAG3_HUMAN Lymphocyte activation gene 3 protein OS = Homo sapiens GN = LAG3 PE = 1 SV = 5) after PMF analysis led to a confident Mascot score (>68) by MS alone. The identification was further validated by MS/MS analysis on peptide *m/z* 1422.69 for which a significant score was also obtained (>60) (Figure 6(D)).

The number of peptides detected and the sequence coverage (SC = 39.6 %, see Appendix 2) are similar to values acquired with more classical analysis obtained by in-gel digestion [17]. This fine correlation and peptide distribution across the LAG3 sequence indicates that the on-chip digestion is efficient and not affected by the biochip interface. Furthermore, the MALDI-MS imaging analysis of the SPR channels provided additional indications for the specificity of SPR and MALDI-MS combination by the peptide distributions of *m/z* 1,422.69 specific to LAG3 and *m/z* 1,697.20 specific to α-RSA. The Pepmix (Peptide Calibration Standard) spot was manually deposited for PMF calibration before launching the MALDI-MS imaging analysis and the peptide distribution of *m/z* 1,296.68 specific to Pepmix was only detected on the spot. The LAG3 peptide was only detected on the α-LAG3 arrays, and the α-RSA peptide only on the α-RSA control array. As illustrated in Figure 6(B), no peptides were detected outside the spots, demonstrating the absence of delocalization and cross contamination between the Biacore arrays after (bio)-chemical treatment and matrix deposition.

It is of major interest to combine instrumentation for increased depth of analysis of biomolecular interactions on the surface of biochips. As examples, spatial orientation of cytochrome b5 self assemblies on gold SPR chips has been elucidated in coupling on-chip TOF-SIMS analysis [18] or SPR and atomic force spectroscopy (AFM) could be performed on the same substrate for the characterization of Protein-DNA and Protein-Protein interactions at macro- and nano-scales [19]. The combination of bioassays and MS has several major drawbacks (the lacks of automation procedures, the robustness of the methods, the difficulties to implement a wide range of applications) [20]. We have previously established an analytical platform devoted to SPR imaging in arrays called SUPRA-MS [12]. The array format of SUPRA-MS is more suited to the study of a panel of biomarkers related to the diagnosis of human diseases and its automation of on-chip biochemical treatments followed by MS readout are consistent with clinical proteomics. In this work, we extend the analytical capabilities to arrays in channels based on a lateral flow device which is particularly efficient for the in depth characterization of biomolecular interactions and the elucidation of protein complexes. For both studies, we showed that it was possible to perform, at the femtomole level, extensive MALDI analysis subsequent to the SPR characterization of protein-protein interactions in human plasma. Particularly, the proposed solution includes all the potentialities of MALDI-MS, *i.e.*, PMF and MS/MS based antigen identifications and the monitoring of on-chip peptide distributions at high spatial resolving power by MALDI imaging. Our results are in the range than those obtained by Yan and co-workers which have developed immuno-MALDI assay on a porous silicon chip and validated it with known amounts of angiotensin I (from 1 to 10 nM) in diluted human plasma [21]. Moreover, it is important to notice that the notion of biomarker does not imply solely the tracking of low abundant proteins in complex media but also the characterizations of variants of abundant proteins, these variants themselves becoming markers as is the case for hemoglobin variants for sickle cell disease [22,23].

## 4. Conclusions

We established here a complete SPR-MS combination compatible with the Biacore biochip format. In this study, we developed a procedure for the detection and the characterization at ∼1 μg/mL of the LAG3 protein spiked in human plasma. The analytical performance of this new method was established, particularly its specificity (S/N > 9) and sensitivity (100% of LAG3 identification with high significant mascot score >68 at the femtomole level). Moreover, SPR-MS offers another vision of captured or immobilized proteins in providing the peptide profiling and the amino acids sequencing.

Affinity enrichment using SPR coupled with the on-chip identification by MS is of major interest in proteomics and diagnosis. For this latter point, the combination of bioassays and mass spectrometry could allow, in a near future, discrimination of protein variants. In this purpose, it is interesting to note the emergence of combination immuno-MALDI MS techniques (as SUPRA-MS, ELISA-MS, SISCAPA…) which have the potential for providing new knowledge on targeted proteins which could become new biomarkers for diagnosis purposes [2,12,24].

## Figures and Tables

**Appendix 1.**
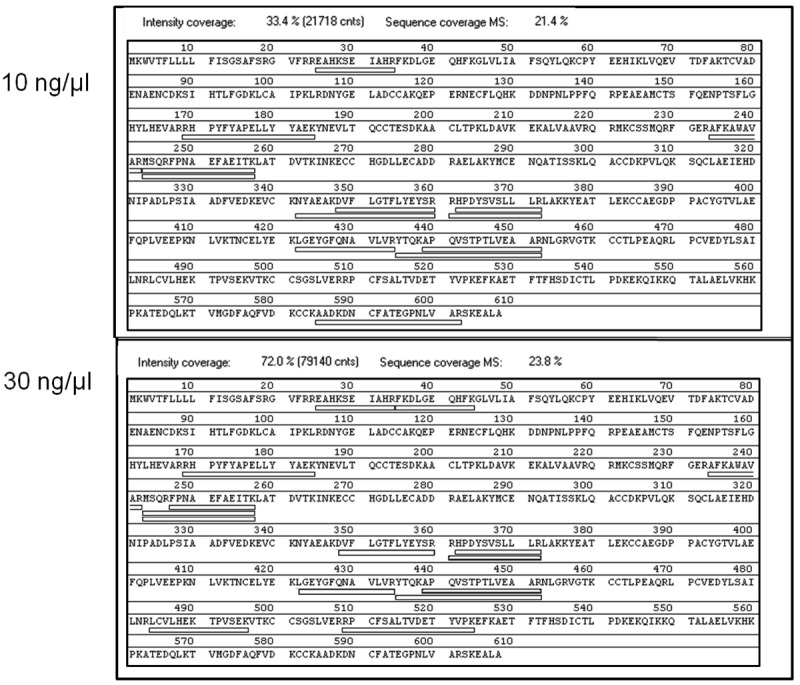
Sequence coverage of the matching peptides detected after reduction and digestion of the deposited RSA at 10 fmol/mm^2^ using 10 and 30 ng/μL of trypsin.

**Appendix 2.**
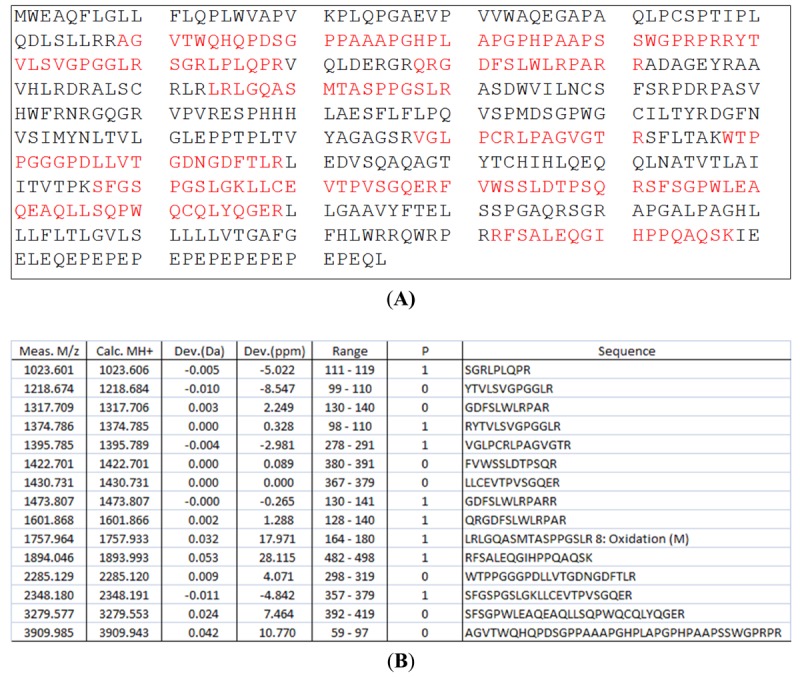
sequence coverage (**A**) and Peptide list (**B**) obtained after biochemical treatments of the captured LAG3 protein in human plasma related to the green array in Figure 6(A,C).
